# Supplementary material for: Investigation on the Microscopic/Macroscopic Mechanical Properties of a Thermally Annealed Nafion® Membrane
Source: Polymers (Basel). 2021 Nov 20;13(22):4018. doi: 10.3390/polym13224018 (PMC8620802; doi:10.3390/polym13224018)
Supplement: Supplementary file 1 [file polymers-13-04018-s001.zip › polymers-1470320-supplementary.pdf]

Supporting Information

Investigation on microscopic/macroscopic mechanical properties of thermally annealed Nafion® membrane

Tuyet Anh Pham[1,†], Seunghoe Koo[1,†], Hyunseok Park [1,†], Quang Thien Luong[2], Oh Joong Kwon[2], Segeun Jang[3], Sang Moon Kim[1,*], Kyeongtae Kim[1,*]

[1] Department of Mechanical Engineering, Incheon National University, Incheon 22012, Republic of Korea

[2] Department of Energy and Chemical Engineering, Incheon National University, Incheon 22012, Republic of Korea

[3] School of Mechanical Engineering, Kookmin University, Seoul, 02707, Republic of Korea

† These authors contributed equally to this work.

***Correspondence**

Sang Moon Kim: ksm7852@inu.ac.kr

Kyeongtae Kim: kyekim@inu.ac.kr

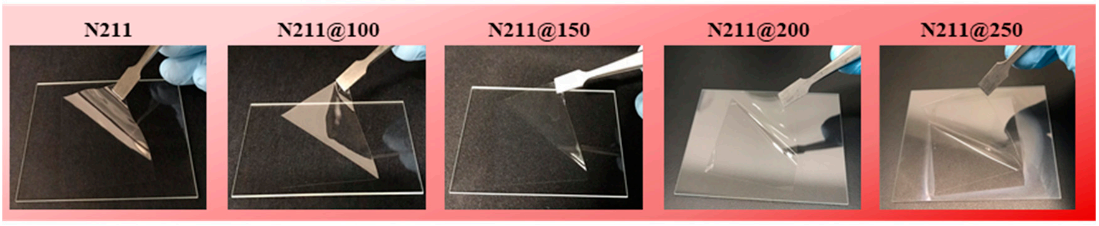

Figure S1. Color change of membrane after annealing.

Calculation of Young's modulus of Nafion membranes

Herein, the two-point method reported by Sun *et al.* was applied to analyze the Young's modulus of the Nafion membranes.

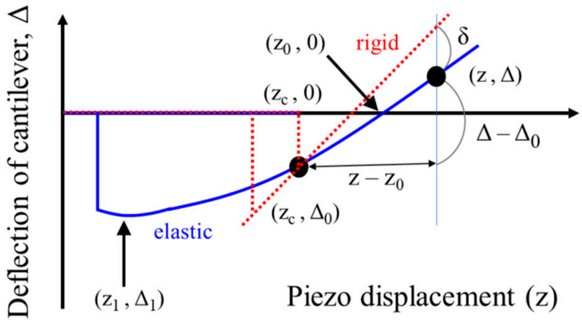

Figure S2. Schematic of deflection–displacement curves between sample and AFM probe. Dashed line in red represents the case of rigid material, and solid line in blue represent the case of soft material [1].

Figure S2 shows the schematic of the deflection–displacement curve. The Piezo displacement in the *x*-axis and deflection of the cantilever in the *y*-axis are plotted in the x–y plane. The dashed line in red is a curve measured with rigid materials (e.g., silicon dioxide), and the solid line in blue is a curve measured with soft materials (e.g., Nafion membrane herein). Since there is no

deformation in rigid materials, the deflection of the cantilever and Piezo displacement are the same. Therefore, the force applied to the sample can be calculated by multiplying the spring constant, k = 2.8 N/m, and the deflection of the cantilever. For soft materials, the deflection of the cantilever is not the same as the Piezo displacement due to the inherent deformation of the materials in response to the applied external stress, which is different from the rigid material. Furthermore, the difference in cantilever deflection for rigid and soft material considering the Piezo displacement is used to calculate the deformation of soft materials (δ), which is expressed as follows:

$$\delta = (z_{trig} - z_c) - (\Delta_{trig} - \Delta_0) \,. \qquad \text{(S1)}$$

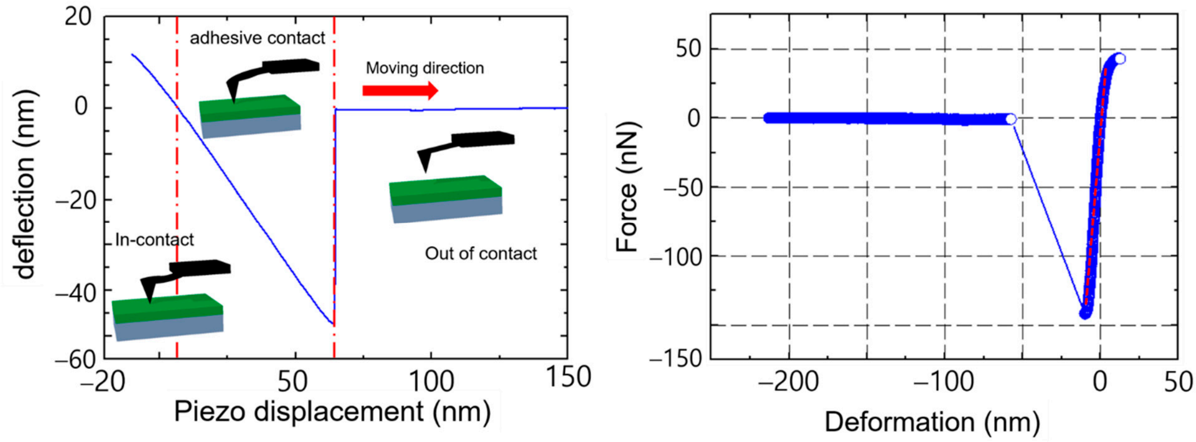

Figure S3. Deflection–displacement curve (left) and force–deformation curve (right). **Figure S3** shows the deflection–distance curve for the Nafion membranes and the force–deformation curve obtained using the above equation. Then, the Young's modulus of Nafion membrane can be calculated by substituting the deformation values (δ_0 and δ_1) in the graph at two points and the Poisson ratio (ν) of 0.4 [2] into the following equation.

$$E = \frac{3(1-\nu^2)}{4} \times \frac{1.27F_1}{\sqrt{R(\delta_0 - \delta_1)^3}} \qquad \text{(S2)}$$

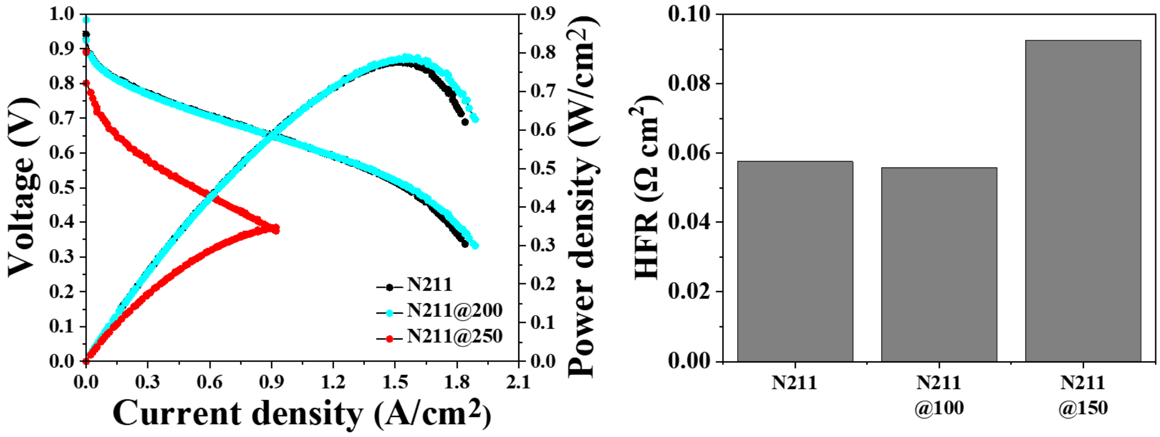

Figure S4. Polarization curves of MEAs with N211, N211@100, N211@150, N211@200 and N211@250.

Table S1. High-frequency resistances of the MEAs and relative ionic conductivity.

Sample	HFR [$\Omega \cdot cm^2$]	Relative ionic conductivity
MEA with N211	0.0576	100 %
MEA with N211@200	0.0559	103.04 %
MEA with N211@250	0.0925	62.27 %

References

[1] Wang, D., Fujinami, S., Liu, H., Nakajima, K., Nishi, T.. Investigation of true surface morphology and nanomechanical properties of poly (styrene-b-ethylene-co-butylene-b-styrene) using nanomechanical mapping: Effects of composition. *Macromolecules* **2010**, 43, 9049–9055.

[2] Almaraz, G. D., Sánchez, R.H., Martínez, A.G., Gómez, E.C., Juárez, J.V., & Garza, V. L. Ultrasonic fatigue tests on the nafion proton exchange membrane, under the modality of three points bending. *Procedia Structural Integrity* **2017**, 3, 571–578.
